# Transabdominal Fine-Needle Aspiration Biopsy: A Colour Atlas and Monograph (Grace Chia-yu Hsu Yang and Liang-Che Tao), Second Edition: a book review

**DOI:** 10.1186/1742-6413-5-11

**Published:** 2008-04-25

**Authors:** Prabodh K Gupta

**Affiliations:** 1 Pathology & Laboratory Medicine, Hospital of the University of Pennsylvania, Philadelphia, PA, USA

## 

Transabdominal fine needle aspiration biopsy has undergone considerable progression since the first edition of this book published by Dr. Tao in 1990. The present monograph has been built upon the foundation laid by Dr. Tao-a pioneer in transabdominal fine needle aspiration biopsy. Dr. Tao was able to bring his extensive personal experience that helped shape the inter-abdominal aspiration cytopathology to a considerable degree in this country. Dr. Grace Yang has further improved this classic by inclusion of her own broad experience in the field, incorporation of newer techniques including liquid-based preparation and development of rapid Ultrafast Papanicolaou stain (UPS). Dr. Yang is credited with this procedure. It is quick and reproducible. Although, not widely used, the procedure is promising and needs some experience and hands on experience for meaningful evaluation and interpretation.

The book is divided into 12 chapters. It starts with a brief history and basic principles of the fine needle aspiration procedures. The third chapter provides an excellent review to the approach of diagnostic cytopathology. It incorporates useful, informative tabulation of the key features helpful in distinguishing various inter-abdominal lesions. The cytopathology of each organ is approached in a logical, systematic way, providing features of the normal structure followed by extensive in-depth presentation of the various morphological features necessary for diagnosis of the underlying pathology. There is a chapter on malignancies of lympho-reticular system. It follows the current World Health Organization (WHO) classification. Similarly current morphological classifications for lesions of liver, pancreas, and kidney have also been provided. The appropriate use of various bio-makers and ancillary techniques is adequately covered; many of these procedures tend to be transitory and may not withstand the test of the time.

True to the Chinese proverb – "A picture is worth a thousand words", this monograph includes over a thousand pictures mostly good quality photomicrographs, some relevant diagrams, radiographs and some ultra-photomicrographs. The CD ROM serves as a stand-alone source of information; it provides high-quality images complementing the book. Dr. Yang and Dr. Tao have compressed an enormous amount of information in this well-illustrated book that should be valuable in the interpretation of intra-abdominal fine needle aspirations. This book is strongly recommended to the Cytopathology community.

